# Supplementary figures and images for: Differential Modulation of Matrix Metalloproteinases-2 and -7 in LAM/TSC Cells
Source: Biomedicines. 2021 Nov 24;9(12):1760. doi: 10.3390/biomedicines9121760 (PMC8698908; doi:10.3390/biomedicines9121760)

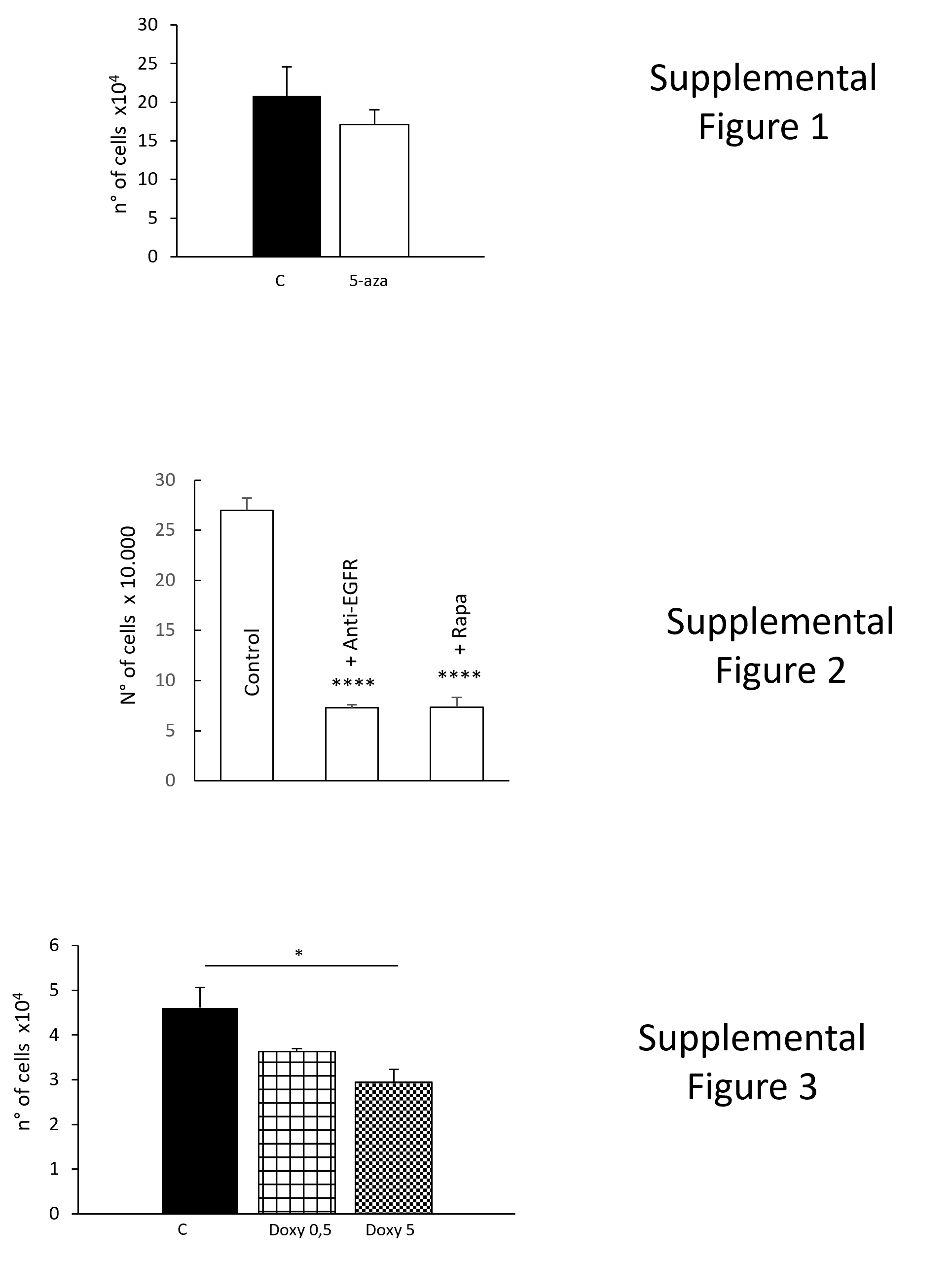

Supplement: Supplementary file 1 [file biomedicines-09-01760-s001.zip › biomedicines-1338137-supplementary.tif]
